# Methods of data collection and analysis for the economic evaluation alongside a national, multi-centre trial in the UK: Conventional ventilation or ECMO for Severe Adult Respiratory Failure (CESAR)

**DOI:** 10.1186/1472-6963-8-94

**Published:** 2008-04-30

**Authors:** Mariamma M Thalanany, Miranda Mugford, Clare Hibbert, Nicola J Cooper, Ann Truesdale, Steven Robinson, Ravindranath Tiruvoipati, Diana R Elbourne, Giles J Peek, Felicity Clemens, Polly Hardy, Andrew Wilson, The CESAR Trial Group

**Affiliations:** 1 School of Medicine, Health Policy and Practice, University of East Anglia, Norwich, NR4 7TJ, UK; 2 Health Economics & Decision Science, School of Health & Related Research, University of Sheffield, S1 4DA, UK; 3 Department of Health Sciences, University of Leicester, LE1 7RH, UK; 4 London School of Hygiene and Tropical Medicine, London, WC1E 7HT, UK; 5 Glenfield Hospital, Leicester, UK, LE3 9QP, UK; 6 Frankston Hospital, Victoria, 3199, Australia; 7 Clinical Epidemiology and Biostatistics Unit, Royal Children's Hospital, Melbourne, Australia

## Abstract

**Background:**

Extracorporeal Membrane Oxygenation (ECMO) is a technology used in treatment of patients with severe but potentially reversible respiratory failure. A multi-centre randomised controlled trial (CESAR) was funded in the UK to compare care including ECMO with conventional intensive care management. The protocol and funding for the CESAR trial included plans for economic data collection and analysis. Given the high cost of treatment, ECMO is considered an expensive technology for many funding systems. However, conventional treatment for severe respiratory failure is also one of the more costly forms of care in any health system.

**Methods/Design:**

The objectives of the economic evaluation are to compare the costs of a policy of referral for ECMO with those of conventional treatment; to assess cost-effectiveness and the cost-utility at 6 months follow-up; and to assess the cost-utility over a predicted lifetime. Resources used by patients in the trial are identified. Resource use data are collected from clinical report forms and through follow up interviews with patients. Unit costs of hospital intensive care resources are based on parallel research on cost functions in UK NHS intensive care units. Other unit costs are based on published NHS tariffs. Cost effectiveness analysis uses the outcome: survival without severe disability. Cost utility analysis is based on quality adjusted life years gained based on the Euroqol EQ-5D at 6 months. Sensitivity analysis is planned to vary assumptions about transport costs and method of costing intensive care. Uncertainty will also be expressed in analysis of individual patient data. Probabilities of cost effectiveness given different funding thresholds will be estimated.

**Discussion:**

In our view it is important to record our methods in detail and present them before publication of the results of the trial so that a record of detail not normally found in the final trial reports can be made available in the public domain.

**Trial Registrations:**

The CESAR trial registration number is ISRCTN47279827.

## Background

Extracorporeal Membrane Oxygenation (ECMO) was introduced into treatment of severe but potentially reversible respiratory failure in the 1970s. The technique involves placing patients on a life support circuit with a membrane oxygenator to temporarily take over the gas exchange function of e lung thereby allowing the lungs to rest and recover [1,2]. The early reports of the use of ECMO in adult with severe respiratory failure were enthusiastic [3]. It soon became clear however, that although ECMO was effective and cost effective compared to conventional ventilation in newborns [4], the evidence was much less clear for the adult population. Many centres in the world use ECMO technology and have reported survival rates in excess of 50% in uncontrolled observational studies of patient outcomes [5,6]. However, considerable improvements have also been reported in survival rates of conventionally treated patients with severe respiratory failure [7-9].

Given the high cost of treatment, ECMO is considered an expensive technology for many funding systems. However, conventional treatment for severe respiratory failure is also one of the more costly forms of care in any health system [10]. Differences in lengths of stay and types of care received by patients following either clinical pathway may result in different statistical distributions of cost for inpatient care. In addition, because appropriate care is provided in relatively few centres, the location of care and need for specialist transport for patients also affects the costs of care. Finally, if there is increased survival to discharge from hospital, then there will be more use of services in primary and community care, and requirement for help for recovering people at home. Thus the health service costs and the household costs might fall at any stage of the treatment and recovery, and in many different forms.

In addition to the costs of alternative forms of care, the economic choice depends on the value of the outcome gained. Uncertainty about the effectiveness of referral to an ECMO centre led to a trial to assess the costs and effectiveness of the new form of care funded by the NHS Health Technology Assessment programme. The protocol for the 'Conventional ventilation or ECMO for Severe Adult Respiratory failure (CESAR) Trial was published in 2006 [11]. This paper provides details of the methods used for the economic evaluation, mentioned in the protocol and conducted as an integral part of the CESAR trial.

### Previous economic evaluations

A literature search failed to find any economic evaluation studies of adult ECMO. However, there have been a series of economic evaluations of ECMO in babies alongside the UK collaborative randomised trial of neonatal ECMO [12] which reported the estimated additional cost (UK 1994–95 price) of ECMO per additional surviving infant with no disability as £75,327 at one year of age. Follow-up at 4 and 7 years for the same study shows the incremental cost (UK 2001 & 2003 price) of neonatal ECMO to be £24,775 & £23,566 per disability-free life year gained [13,14]. Similarly a retrospective cost-utility analysis [15] reports costs of USD 24,386 per quality adjusted life-year saved for 'salvage ECMO' in children. In all cases, in spite of the high cost of neonatal ECMO, the incremental cost per QALY was within health care funders' range of acceptable value for money. This remains a question in the case of adult ECMO.

### The CESAR Trial

The CESAR trial [11] was designed to compare two alternative strategies for treating severe but potentially reversible respiratory failure: conventional ventilation, and transfer to a centre providing ECMO. In the UK, during the CESAR trial, ECMO is provided by Glenfield Hospital, Leicester, and conventional treatment by other UK hospitals capable of providing a high standard of care for ECMO eligible patients.

The primary outcome measure for the clinical evaluation is increase in survival at 6 months without severe disability ('confined to bed' and 'unable to wash or dress') at six months. Power calculations based on estimates of these outcomes from severe adult respiratory distress syndrome (ARDS) suggested a sample size of 180 would have sufficient power to detect a reduction in primary outcome by a third (based on 5% statistical significance, 2-sided test and 80% power). All ICUs in the UK were invited to take part in the trial and 148 units referred patients for consideration for entry to the trial. The participation of so many ICUs is necessary due to the small numbers of adults who suffer from the condition annually.

## Methods

### Economic questions about treatment of severe respiratory failure

The economic evaluation addresses the question of value for money of the alternative treatment options. The economic question asks "for patients with severe but potentially reversible respiratory failure, is ECMO cost-effective from the viewpoints of the NHS and society?" This question can be rephrased "is the additional cost of achieving an important gain in outcome within the range that the health funding system, or society, is willing to pay?"

The objectives of the economic evaluation are:

• To compare the costs of a policy of referral for ECMO with those of conventional treatment.

• To assess the cost-effectiveness of referral for ECMO compared with conventional treatment in terms of additional survival with and without disability at six months post-randomisation.

• To assess the cost-utility of referral for ECMO compared with conventional treatment in terms of utility gain as measured by EQ5D at 6 months follow-up.

• To assess the cost-utility of referral for ECMO compared with conventional treatment in terms of utility gain as measured by EQ5D, and other sources, over a predicted lifetime.

### Design of the Economic Evaluation alongside the CESAR Trial

The design of this economic evaluation alongside the CESAR trial is based on published recommendations [for example, [16]]. This involves defining: the type of economic evaluation to be conducted; the comparator form of care; the perspective and time horizon for costs and outcomes; appropriate outcome measures for each perspective and type of evaluation; identification, measurement and valuation of resources; estimation of unit costs; and a plan for economic analysis, which includes decisions on discounting future costs and consequences, tackling uncertainties and presentation of results.

#### Type of economic evaluation

The first planned analysis is a cost effectiveness analysis (CEA) with increase in survival without severe disability at six months (the primary outcome in the CESAR trial) as the main outcome measure. A short term cost utility analysis (CUA) was also planned in which health benefits are quantified in terms of quality-adjusted life-years (QALYS), and measured using the instrument EQ-5D at 6 months. Lifetime CUA is planned using a decision model based on CESAR trial results and including additional data for predicted lifetime QALYs and health care costs.

#### Comparator

The ideal comparator for any economic evaluation designed to assess the cost effectiveness in a particular context is the most commonly used treatment for the condition in that context. The CESAR trial was designed as a pragmatic comparison, where patients allocated to conventional care were receiving treatment that would be the normal form of care in the NHS. To ensure that the patients in the control group received as near as possible the best practice of care, the CESAR trial protocol specified aspects of service provision that must be considered, including facilities available at the participating ICUs, experience of treating such patients, and certain aspects of the clinical treatment protocol for ventilated patients. Full details are given in the CESAR trial protocol [11]. In general, however, the comparator group was intended to be representative of NHS care provision (in qualifying ICUs) for acute respiratory failure during the period of the trial.

#### Perspective or viewpoint for analyses

In the UK, the National Institute of Health and Clinical Excellence (NICE) proposes that applicants presenting economic analyses for NICE appraisals should take a NHS perspective [17]. However, there are aspects of public patient choice and valuation that may not be considered in such an analysis. Economic evaluators are guided to take a societal viewpoint if possible [16]. As the ECMO technology may be adopted for review by NICE or a similar agency in the UK, it was decided that the perspective for the CESAR trial should include both the NHS and societal perspectives. The latter viewpoint is important, as the results of this study are likely to have economic impacts other than through health care requirements if there is significantly increased survival of either able bodied or disabled adults. It is also anticipated that the results of the trial may provide useful information for a wider international audience where different ranges of services are provided within the health system.

#### Time horizon for economic evaluation

The follow-up duration for the CESAR trial is 6 months. This does not allow the full long term cost and benefits to be measured. However, it satisfies the recommendation of the American Thoracic Society for cost-effectiveness analyses of ICU therapies to have a minimum follow-up period of 6 months [10]. However, to meet our fourth objective, prediction and modelling long-term (lifetime) costs and benefits are also planned.

### Outcome measures for economic evaluation

#### Survival without severe disability

Death of patients in the trial was recorded during the period of follow up whenever it occurred. Staff at the CESAR trial data management centre maintained contact with all centres with patients being treated within the CESAR trial ensuring complete reporting. For those discharged from hospital, contact was sought either through their home, or through their family doctors, if patients consented to be approached in either of these ways. Any further deaths would be reported in this way. Severe disability in survivors at six month was defined as those who were unable to care for themselves and were confined to bed: that is who had worst possible scores for the Euroqol EQ5D domains for self care and for mobility.

#### Quality adjusted life years (QALYs)

The calculation of QALYs was planned to be based on assessment of health related quality of life at six months from randomisation. The EQ-5D is a standardised instrument used for measuring health outcomes. Quality adjusted health utility weights for each patient are calculated for the CESAR trial using UK specific utility values for each patient's response to the EQ5D at 6 months. We could find no previous models for estimation of QALYs gained at 6 months in similar patients, and so they are estimated assuming that the value of the health state at trial entry was zero, and that over the months of survival, patients have experienced linearly increasing quality of life up to the level at 6 months.

Estimates of lifetime QALYs are predicted based on assumptions of gradual improvement of quality of life up to 2 years from randomization [18-22], and of predicted life expectancy based on age specific rates for the population of England and Wales. Age and sex specific life expectancy is calculated for each surviving patient in the trial using UK life tables [23]. It is assumed that, at 24 months post randomization, all surviving trial patients attained the same average life expectancy and health state as adults of similar age in the UK population. It is assumed that average health states for different age groups would be the same as those obtained from the 1996 Health survey for England [24].

### Cost estimation

#### Identifying resource use

For the CESAR trial relevant aspects of resource use were identified using expert advice (managers, medical, nursing and patient representatives all commented on the draft lists) and also considering the items included in the economic evaluation of neonatal ECMO [12]. A list of resource items important from one or more viewpoints is given in Table 1. This includes resource use associated with initial stay in intensive and high dependency care units at different levels of care (measured by number of organs supported – see below), use of ambulance transport, stays in other hospital wards before discharge, costs of visiting incurred by relatives whilst patients are in hospital, resource use after discharge up to six months, major changes in household, out-of-pocket expenses of patient and family, loss of paid and unpaid working time, changes in working time, and informal care.

**Table 1:** Items of resource use in the CESAR trial

**Resource items**	**Instrument for data collection within CESAR trial**	**Source of unit cost data**	**References to sources**
**From trail entry to discharge from hospital**			
Days of organ support	Daily organ support form	ICU costing study	[36,37]
Days on ECMO	Daily organ support form	ICU costing study	[36,37]
Days on conventional ventilation	Daily organ support form	ICU costing study	[36,37]
Days in intensive care	Daily organ support form	ICU costing study	[36,37]
Days of other hospital stay before discharge	Outcomes data sheet	PSSRU –	[25]
Miles transported by air ambulance	Transport forms (a) and (b)	cost provided by transport provider	
Miles transported by land ambulance	Transport forms (a) and (b)	cost provided by ambulance trusts	
**From discharge to follow-up at 6 months**			
Telephone contacts with GP	Events diary and patient cost questionnaire	PSSRU	[25]
Contacts with NHS direct	Events diary and patient cost questionnaire	NHS direct personal communication	
Visits to GP	Events diary and patient cost questionnaire	PSSRU	[25]
Home visits by nurse	Events diary and patient cost questionnaire	PSSRU	[25]
Visits to counsellor	Events diary and patient cost questionnaire	PSSRU	[25]
Visits to physiotherapist	Events diary and patient cost questionnaire	PSSRU	[25]
Visits to occupational therapist	Events diary and patient cost questionnaire	PSSRU	[25]
Visits by health visitor	Events diary and patient cost questionnaire	PSSRU	[25]
Days of inpatient stay	Events diary and patient cost questionnaire	PSSRU	[25]
Outpatient visits	Events diary and patient cost questionnaire	PSSRU	[25]
A&E visits	Events diary and patient cost questionnaire	PSSRU	[25]
Visits to day hospital/day care	Events diary and patient cost questionnaire	PSSRU	[25]
Days in residential care	Events diary and patient cost questionnaire	PSSRU	[25]
Days in nursing home	Events diary and patient cost questionnaire	PSSRU	[25]
Medication	Events diary and patient cost questionnaire	PSSRU	[25]
Visits by social worker	Events diary and patient cost questionnaire	PSSRU	[25]
Visits by homecare worker	Events diary and patient cost questionnaire	PSSRU	[25]
Aids & adaptations	Events diary and patient cost questionnaire	Reported by participants and some estimated from personal enquiries by researcher to equipment suppliers	
Value of hours of informal care	Events diary and patient cost questionnaire	ONS	[30]
Miles of private car use for health care	Events diary and patient cost questionnaire	Automobile Association (AA)	[28]
Out-of-pocket expenses	Events diary and patient cost questionnaire	Reported by CESAR trial patients	
Major changes in household	Events diary and patient cost questionnaire	Reported by CESAR trial patients	
Childcare costs	Events diary and patient cost questionnaire	Reported by CESAR trial patients	
Change in employment	Events diary and patient cost questionnaire	Reported by CESAR trial patients	
Change in benefits or allowances	Events diary and patient cost questionnaire	Reported by CESAR trial patients	
Loss of income from employment	Events diary and patient cost questionnaire	Reported by CESAR trial patients	
Other costs	Events diary and patient cost questionnaire	Reported by CESAR trial patients	
Other changes	Events diary and patient cost questionnaire	Reported by CESAR trial patients	

#### Measuring resource use

Resource use data are collected prospectively for every trial participant at various points of his/her progress from recruitment to follow-up using a series of data forms and questionnaires. Some, but not all, of these are additional to the instruments planned for the CESAR trial management and clinical outcome data collection [11]. These instruments are:

a) Daily organ support form – completed by intensive care staff for each trial participant on a daily basis, and used to classify intensity of resources used during the intensive care ECMO/conventional treatment period.

b) Transport form (a) at trial entry – completed by Glenfield Hospital transport team to record transfer of trial participants to ECMO centre or conventional treatment centres.

c) Transport form (b) – completed by Glenfield transport team to record ambulance journey of participants returning either to the original recruiting hospital or another intensive care unit after ECMO.

d) Outcomes data sheet – completed by medical staff and records date on death of patient (if applicable), date of discharge, date of transfer to another hospital/home, use of ambulance for transfer etc.

e) Events Diary -to be completed and kept by every participant to document all services used from discharge to follow-up as an *aide memoire *to help them to answer questions at 6 months. This included information about informal help received as well as formal services.

f) Patient cost questionnaire at 6 month follow up – administered by trained interviewer at patient's home or by telephone to collect resource use data from discharge to follow-up, covering items recorded in (e) above.

g) GP proforma – completed by GPs to collect medication use of those patients who refuse the 6-month follow-up but give permission for use of GP records.

The Events Diary (e) and the Patient cost questionnaire (f) were piloted with five patients discharged from Glenfield Hospital ICU, and the GP proforma (g) piloted with 5 general practitioners. Interviewers were trained in the administration of the patient cost questionnaire (f). As it was anticipated that many Ambulance Trusts across UK may become involved in transporting trial patients, all ambulance trusts were contacted and agreement obtained to provide costs of patient journeys (including overhead & running costs) as and when it took place during the trial.

Two items of resource use not collected alongside the trial are: resource use associated with and following a patient's death in ICU, and cost incurred by relatives whilst visiting patients in intensive care/hospital stay. These items were excluded from the data collection from CESAR trial patients due to the practical difficulty of collecting data and due to the lack of a well-defined methodology available at the early stages of planning the CESAR trial. However, the cost of visiting patients in intensive care was thought likely to be an important social cost, and is being estimated by a separate study in a sample of CESAR centres and is described in more detail under "estimating unit costs" below.

#### Resource data collection for the economic evaluation

Following recruitment, the progress of all participants is tracked initially until their discharge from hospital so that resource use, and clinical progress, can be accurately measured and collected at each stage. During the intensive treatment period (ECMO or conventional ventilation) data are collected on number of days spent in each treatment mode, including daily information on number of organs supported and the level of critical care (ICU or HDU). After transfer to another hospital or another ward within the same hospital after the acute phase of the illness, resource use is measured as number of in-patient days up to discharge.

Details of all ambulance use related to transferring trial patients at recruitment are collected by the Glenfield transport team and details of all other ambulance journeys (for example transfer between hospitals) are collected by the relevant hospitals and sent to the research team. Data collected include date, time, origin and destination of journey, mode of transport (road ambulance, fixed wing aircraft, or helicopter), duration of journey, and distance travelled by patient.

After discharge from hospital, each participant is sent details of the forthcoming interview and the 'events diary' to record resource use. The patient is asked to give permission for one of a series of options to take place six months after trial entry: (1) face-to-face interview (2) telephone interview (3) postal questionnaire and (4) collection of resource use from GP records. Those patients still in hospital at six months if fit enough are asked to give permission to be interviewed at their hospital bedside using a very short resource use questionnaire.

#### Estimating unit costs

In order to estimate total cost of treatment for each trial participant, the respective quantities of resource use are multiplied by their corresponding unit costs. Some resources used by participants are in the form of actual costs (not charges) and do not need any valuation. For example, cost of ambulance journeys are obtained directly from the relevant ambulance service providers and incorporate all overhead and running costs. The unit costs of most items of resource use are obtained from nationally available sources [25,26]. Use of medication is valued using the price of drugs listed in the *British National Formulary *[27]. Informal care is valued by the opportunity cost method suggested by Posnett & Jan [28]. Average cost per day of ICU and ECMO is obtained from a separate study and weighted/adjusted for each centre in the CESAR trial (see "*Cost/day of ICU including ECMO unit care" *below). Cost of visiting is also derived from a separate study (see "*Costs of visiting patients in intensive care" *below). Costs of private travel will be estimated using Automobile Association (AA) [28] motoring costs.

#### Valuation of informal care time

Informal time will be valued using weights suggested for Posnett & Jan's [29] scenarios: working time were output is replaced; working time where output is not replaced; non-work time of those in paid employment and those not in paid employment; and finally time for those not in paid employment where unpaid housework is not replaced. Average wage rates of men and women in the United Kingdom needed for estimating time costs is obtained from Office of National Statistics (ONS) [30].

#### Predicted future costs of lifetime care

It was assumed that survivors at 6 months would continue to have similar average daily costs of care as at the 6 months follow up point, until 24 months post randomization. At 24 months, the average health service expenditure for the surviving patients in the CESAR trial was assumed to be the same as that of similar age groups in the UK. The age groups used in predicting future costs and benefits were: 16–44 years, 45–64 years, 65–74 years and 75–84 years. Data on health services costs for these age groups have been published in the proceedings of Parliament [31]. The same age groups were used as the basis for estimating both patients' long-term costs and their benefits.

#### Price year, inflation, currency and discounting

Resources and costs will be measured in the year in which they occur using appropriate unit costs for each year of resource use. All costs are then revalued for analysis and reporting to 2005 UK values using health care inflation estimates.

The follow-up duration for the short term analyses is 6 months and therefore discounting is not necessary. For the lifetime estimates, costs and QALYs were discounted at 3.5%, based on UK Treasury guidelines [32].

### Cost per day of ICU including ECMO unit care

The task of achieving a case-mix adjusted daily costs of ICU care was achieved through a prospective, observational, longitudinal multi-centre study (the 'Critical Care HRG study'), concurrent with the CESAR trial, involving a volunteer sample of 70 critical care units, where monthly data on critical care unit expenditure together with daily data on patients' organ support were collected for a two/three-month period [33]. The sample of participating critical care units had good geographical coverage in England with smaller numbers from Scotland and Northern Ireland, but none from Wales. An average daily cost of ICU was estimated by collecting data on the monthly expenditure of intensive care units and apportioning this sum by their monthly throughput of patients. Case-mix adjustment of this average daily cost was achieved by a weighting based on the number of organs supported on that day.

#### Data collection

Data on patients' organ support requirements were collected on a daily basis by the critical care unit staff using specially designed data collection booklets. These data were collected for consecutive admissions during the study period. At the same time, the intensive care units and hospital finance departments were sent questionnaires to document their monthly expenditure on consumables (drugs and fluids, disposable equipment, nutritional products and blood and blood products), staff (consultant medical staff and other medical staff), clinical support services (radiology tests and laboratory services), professionals allied to medicine (physiotherapists, clinical pharmacists, dieticians, medical technical officers, information technologists, clinical and biomedical scientists, speech and language therapists, clinical psychologists and occupational therapists), support staff (personnel officers and directorate accountants) and specialised bed therapy. Data were also collected on the organizational characteristics of the intensive care units and the monthly number of patient days, number of staffed beds, number of patient admissions etc. An average daily cost was calculated using the following formula:

$$\frac{\Sigma \text{ Monthly expenditure on Staff}+\text{Consumables}+\text{Clinical Support Services}}{\text{Monthly number of patient days}}$$

The average daily cost in critical care ICU had to be adjusted to reflect the severity of illness or degree of organ support required by patients. For this purpose, data provided by 46 critical care units in the Critical Care ICU HRG study [34] were used. Only those critical care units that supplied data on their expenditure, organ support and unit characteristics were included in this analysis. The aim was to develop an appropriate model from which estimates of daily case-mix adjusted costs could be determined.

Different ways of modelling the organ support and expenditure data were explored. The model of choice was informed by the Breusch-Pagan and Hausman specification tests [35] that favoured a random-effects model based on the number of organs supported on a daily basis; clustered to include 0–1 organ, 2 organs and = 3 organs. This model offered a simple and reproducible system of estimating case-mix adjusted costs of care. Daily organ support weights were 0.577 for 0–1 organ supported, 1.137 for 2 organs supported and 1.156 for = 3 organs supported [36]. These weights will be applied to average daily costs of patients participating in the CESAR trial. A total cost per patient of their ICU stay was calculated by weighting patients' average daily cost according to the number of organs supported on a daily basis and summing these daily costs for each patient.

Internal validation of the average daily cost data collected was not performed, however external validation was possible using data collected by the Critical Care National Cost Block Programme [37]. Twenty-one intensive care units in this study (30%) contributed data to the Cost Block Programme for the financial year 2000–2001. Although the Cost Block Programme collected data for a different time period and using a different configuration of units, the similarity between the mean costs per patient day is striking, in particular, the costs of consumables and clinical support services. The study by Hibbert et al [33] had wider coverage of resources with respect to professionals allied to medicine and an in-built allowance for capital equipment, which may be responsible for a slightly higher mean costs per day (£1302, 2003 price year) compared to £1028 (2001 price year, £1119 inflated to 2003 price year) for the Cost Block Programme.

The completeness of the returned data was investigated by each resource item and expressed as a percentage of the number of responses divided by the total number of 18 possible responses which reflected the quantity of data sought from participating centres. Data on nursing and administrative staff together with drugs and fluids yielded the highest number of responses (77%). Data on clinical and biomedical scientists and clinical psychologists yielded the lowest number of responses at 14%.

Not all CESAR centres participated in the Critical Care HRG study. Separate visits or contacts by correspondence were made with all CESAR centres that did not participate in the ICU HRG costing study, including the ECMO centre, to collect the same expenditure data in order to estimate the daily cost in the same way. Forty hospitals recruited patients up until the 31^st ^March 2005. Given that more than one hospital recruited, in some cases, more than one patient during each financial year and patients could have received treatment in both an ICU and an HDU, one hundred and sixteen cost questionnaires were sent out in total to account for this (58 for the ICU and combined ICU/High Dependency Units (HDUs) and 58 for the separate HDUs – where provided). The types of critical care units i.e. which of the participating critical care units had both an ICU and an HDU or operated as a combined ICU/HDU, were not known, so each critical care unit was sent two cost questionnaires for each financial year when a patient was recruited to the trial. Thirteen hospitals completed the expenditure questionnaires however, only 11 hospitals returned data on both their unit characteristics and expenditure, which were needed in order to apportion the expenditure data correctly (i.e. down to an average daily cost). In order to estimate average daily costs for each CESAR hospital for the financial year in which a patient/patients were treated, missing data were substituted with mean estimates obtained from the responding hospitals by financial year.

**Figure 1:**
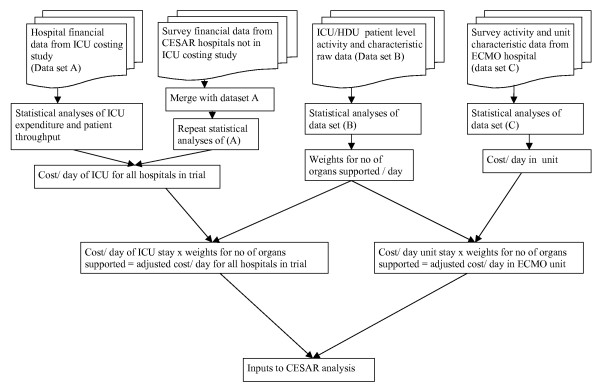
Unit cost flowchart for hospital critical care.

Figure 1 shows the whole process of estimating unit costs of ICU stay, derivation of weights for number of organs supported and how this feeds into the cost estimation in the trial. A fuller description of this part of the research is included in Clare Hibbert's PhD Thesis [36].

### Costs of visiting patients in intensive care

A pilot study of the costs of visiting [38] was carried out in December 2001 at an ICU in the UK. The daily costs per visit estimated in the pilot study are shown in Table 2. The pilot study informed the methods for a multi-centre study in six intensive care units in the UK which are registered with the CESAR trial. The aim was to estimate the average cost of visiting patients in intensive care. All adults including primary carers visiting the intensive care units during a three-week duration were requested to complete a questionnaire that asked them about their time spent in visiting and travel, out-of-pocket expenses, employment status, loss of income etc. Data from this study will be used to estimate the average cost of visiting per day.

**Table 2:** Cost of time foregone, lost pay, out-of-pocket expenses per visit to ICU at UK 2005 prices

**Daily costs**	**Range (£)**	**Mean (£)**	**Median (£)**
Lost pay (n = 5)*	17.36 – 65.10	50.72	54.72
Cost of time foregone (n = 54)	5.04 – 208.32	46.21	24.06
Out-of-pocket expenses	0.00 – 509.54	29.30	9.39

Source: Thalanany et al [38]

### Analysis and reporting of costs and economic evaluation

#### Estimation of costs for each patient

Costs falling upon the health sector (health & social services), upon patients or their families, and other costs such as help from friends will be presented in total and disaggregated form. Resource use and unit costs described above will be used for to estimate mean, medians, standard deviations and ranges of costs for each patient in the CESAR trial.

#### Cost effectiveness analysis

Incremental cost-effectiveness ratio (ICER)

With the availability of patient level data on costs and effects it is possible to summarize uncertainty in the ICER as a confidence interval. As cost data are typically not normally distributed, non-parametric bootstrapping will be used to generate confidence intervals.

#### Cost utility analysis

Lifetime incremental cost-utility ratios will be estimated using bootstrap estimation methods [39,40], and using data and simplifying assumptions described in previous paragraphs.

#### Sensitivity analysis and uncertainty

Sensitivity analysis based on testing specific assumptions and probabilistic analysis will be used to explore the uncertainty in the results [41,42]. Items to be tested in sensitivity analyses are listed in Table 3. Primary analysis will be on complete case basis, where a complete case is defined as cases meeting the CESAR trial clinical effectiveness data analysis. Estimation of the key cost variables is based on between 40 and 50 data items representing different aspects of resource use from each participant. If any single item is missing, the cost variable will also be incomplete. We predict that the complete case analysis will contain a small proportion of the total number of trial participants and thus have a high potential for bias and imprecision. Any missing resource item values will be replaced with imputed values and re-analysed as part of the sensitivity analysis. Missing data will be imputed using Rubin's multiple imputation method [43] with SOLAS v3.20 (Statistical Solutions Inc, Co Cork, Eire).

**Table 3:** Items to test during sensitivity analysis

	**Ranges and thresholds**
Days on ECMO	Highest & lowest observations
Length of stay in Critical Care Unit (ICU & HDU)	Highest & lowest calculated costs
Total length of stay in hospital	Highest & lowest calculated costs
Cost per day on organ support	Highest & lowest calculated costs
Distance from ECMO centre (cost of transport)	Replacing air with road transport
Change in difference in survival	Upper & lower CI of the attributable benefit
Other items with significant cost difference	Highest & lowest observations
Assumption of linear increasing utility for survivors over first 6 months	Assume constant utility at 6 month reported rate

#### Generalising the results to different settings

It would be beneficial to health care decision makers if economic study results could be generalised from one setting to another as this would avoid having to repeat every study in every setting. Factors which may vary in different settings are: unit costs of resources, geographical variations in demography or epidemiology of disease, clinical practice patterns, incentives to health care professionals and availability of resources. To facilitate estimation of the transferability of economic data from the CESAR trial to other health care setting, such factors in the study population will be described, and resource use and prices reported separately.

## Discussion

The CESAR trial is the first RCT of adult ECMO with an economic evaluation incorporated into the design of the trial. The CESAR Trial was funded with full economic support from the design stages of the trial with funding for two part-time health economists which helped the economic research team to tackle many challenges in the design, methods, data collection, developing and piloting the economic questionnaire and planning the analysis. The trial protocol was developed in collaboration with health economists, who are members of the Trial Steering Committee, and an economics working group oversees the economic data collection and analysis.

Incorporation of economic evaluations within randomised controlled trials of medical therapies has been a growing trend in the past decade. Many health care systems in developed countries now use economic evaluations as a formal input to decisions about whether to fund new technologies. In the UK, economic evaluations play a key role in the technology appraisal process at the National Institute of Clinical Excellence (NICE) which makes decisions about a range of health technologies (NICE 2004).

Economic evaluations conducted alongside randomised trials are meant to inform decision-makers about the economic benefit of the technology under investigation. The information will shed the most light on the question of 'value for money' if the trial and the evaluation are properly designed, if appropriate data are collected and correctly analysed, and if the many sources of uncertainly surrounding these evaluations are adequately addressed. The past decade has seen a large increase in the number of published economic evaluations as well as improvements in economic evaluation techniques. However, much debate and confusion still persist among analysts, readers, and policy- makers concerning methods and the overall usefulness of CEA in resource allocation decision making. A number of potential reasons may account for this, among them political expediency, social preferences and systemic barriers to implementation. In addition, there are a number of more technical shortcomings associated with the generation of economic evidence including methodological inconsistency across completed economic evaluations and the limited generalisability or transferability of findings or settings beyond the location of the original study.

The economic evaluation methodology described in this paper aims to address these issues and guidelines and recommendations from more recent publications in methods for economics and trials [44] were used in the design and conduct of the evaluation and the planned analysis.

The CESAR Trial was funded with full economic support from the design stages of the trial with funding for three part-time health economists which helped the economic research team to tackle many challenges in the design, methods, data collection, developing and piloting the economic questionnaire and planning the analysis. The trial protocol was developed in collaboration with health economists, who were members of the trial steering group, and an economics working group including the trial manager and leaders have overseen the economic evaluation.

The strengths of the trial on which this economic evaluation was based are that it was randomised and controlled, pragmatic in design, and provided a vehicle for collecting a comprehensive set of data on resource use and clinical effectiveness. These provide a reliable basis for estimating the economic efficiency of ECMO for adults with severe respiratory failure. The study cost accounting was comprehensive and included most major health service cost items. Most unit costs used for valuation of reported resources used were from published national sources and where unit costs were unavailable rigorous methods were used for their estimation and the methods used clearly described. Unit costs for ICU stays were estimated for every centre that recruited a patient which was then weighted for each patient to reflect the level of care and number of organs supported during the acute phase of the illness. Very few resource items were excluded from the data collection process alongside the trial.

Presenting this methodology paper before the end of the trial is an attempt to make transparent the methods used for the evaluation, and to allay concern of manipulation of economics results. In our view it is important to record our methods in detail and present before publication of the results of the trial so that a record of detail not normally found in the final trial reports can be made available in the public domain.

There are aspects of the planned methods that may be seen as idealistic. In particular, our estimation of resource use after hospital discharge is based on patients' reports after a traumatic period in their lives of many different aspects of service use and personal costs. The aggregate cost variables are made up from a combination of this large number of reported items, many of which may be missing. Although complete case analysis is our primary method of analysis, we are conscious that this might be quite unrepresentative of the CESAR trial population. Our planned secondary approach is to use imputation of missing values to increase the numbers of patients for whom we can estimate costs. However, this also raises the question about how much detail we actually needed to collect from patients (or other sources). Previous researchers have attempted to establish reduced form resource use data for costing [45,46] but have not arrived at any general rules for doing this. Subject to Steering Group approval, the data from this trial will be available for further analysis of this problem.

## Conclusion

As a result of this publication of the methods for the economic evaluation in the CESAR trial prior to publication of the results, we shall be open to scrutiny for any changes to protocol in our reported data collection and analysis. By this means we hope to increase confidence in the results of the economic evaluation.

## Abbreviations

CEA: Cost Effectiveness Analysis; CESAR: Conventional Ventilation or ECMO for Severe Adult Respiratory Failure; CUA: Cost Utility Analysis; ECMO : Extracorporeal membrane oxygenation; ICER: Incremental Cost Effectiveness Ratio; ICU:Intensive care unit; NHS: National Health Service; ONS: Office of National Statistics; PSSRU: Personal Social Services Research Unit; RCT; Randomized controlled trial; UK:United Kingdom.

## Competing interests

The authors declare that they have no competing interests.

## Authors' contributions

**Thalanany MM **– 1) made substantial contributions to conception and design of the economic evaluation in the CESAR trial 2) design of all economic questionnaires, 3) transport data collection 4) design and analysis of the cost of visiting study, 5) was involved in drafting the manuscript and 6) has given the final approval of the version to be published.

**Mugford M **– 1) responsibility for leading and co-ordinating all activities of the economic group, 2) made substantial contributions to conception and design; 2) was involved in revising the manuscript critically for important intellectual content; and 3) has given final approval of the version to be published.

**Truesdale A, Elbourne D, Peek G, Clemens F, Cooper N, Hibbert C, Wilson A **– 1) made substantial contributions to conception and design; 2) were involved in revising the manuscript critically for important intellectual content; and 3) have given final approval of the version to be published.

**Robertson S**, **Hardy P, **– 1) were involved in revising the manuscript critically for important intellectual content, 2) took part in data collection and analysis and 3) have given final approval of the version to be published.

**Tiruvoipati R **– Was involved in revising the manuscript critically for important intellectual content, 2) took part in data collection and 3) have given final approval of the version to be published.

## Pre-publication history

The pre-publication history for this paper can be accessed here:


